# Brace for turbulence: EU Member States’ climate strategies in the aviation sector

**DOI:** 10.1007/s44168-022-00018-1

**Published:** 2022-09-05

**Authors:** Nicole M. Schmidt, Paul Tobin, Brendan Moore

**Affiliations:** 1 Department of Political Science, Heidelberg University, 69115 Heidelberg, Germany; 2 Department of Politics, University of Manchester, Manchester, M13 9PL UK; 3 Institute for European Studies, Brussels School of Governance, Vrije Universiteit Brussel, Pleinlaan 2, Brussels, 1050 Belgium

**Keywords:** Aviation, Climate change, European Union, European Green Deal, Flight, Climate-change policy

## Abstract

The European Green Deal (EGD) aims to transform the European Union (EU) into a climate neutral continent by 2050, requiring significant changes within carbon-intensive sectors. In this article, we examine EU Member States’ climate strategies in the aviation sector, where greenhouse gas emissions have doubled since 1990. We analyse EU National Energy and Climate Plans (NECPs) in order to examine national commitments on aviation. How frequently do EU Member States refer to aviation in their NECPs, and how can these references be understood thematically? First, by conducting a content analysis on all 54 draft and final NECPs, we identify how frequently 11 aviation-related terms were used. While all states increased the number of aviation references between their draft and final NECPs, Central and Eastern states showed the smallest increase. We show that Southern and Western Member States reference aviation most frequently in their final NECPs. Second, in our thematic analysis, we identify three key themes—‘contextual information’, ‘public policy’, and ‘fuels and propulsion’—that we divide into 17 sub-themes. Approximately half of the themes focused on contextual information, such as current aviation emissions, with the other half focusing on carbon reduction strategies, especially the EU Emissions Trading System, biofuels, and taxation. Western EU states provided the largest number of concrete policy statements on reducing aviation emissions and the promotion of biofuels. There were only two references to reducing the number of flights, across almost 7500 pages of climate documents. We conclude that the NECPs represent a promising means of conducting cross-country climate policy analysis, in order to compare efforts across economic sectors within Europe. With our analysis, we create a pre-pandemic sectoral baseline for aviation which can be used for further study.

**Supplementary Information:**

The online version contains supplementary material available at 10.1007/s44168-022-00018-1.

## Introduction

Tackling greenhouse gas (GHG) emissions in the aviation sector will play a crucial role in mitigating climate change. Klöwer et al. (2021) show that aviation contributes around 4% to anthropogenic global warming and will likely cause approximately 0.1 °C of warming by 2050. However, the international, borderless nature of the sector makes the accounting of GHG emissions from aviation challenging; indeed, aviation and shipping are the only two sectors not directly considered within the national targets underpinning the 2015 Paris Agreement (Efthymiou and Papatheodorou 2019). Halting aviation’s contribution to further warming would theoretically be possible by a sustained annual decrease in air traffic by 2.5%, or a transition to a 90% carbon-neutral fuel mix by 2050 (Klöwer et al. (2021). The COVID-19 pandemic helped in this regard as it caused airline emissions to fall by one-third in 2020; but overall, the aviation sector is still not on track to contribute to net zero (International Energy Agency 2021). To date, the aviation industry has preferred a technology-based approach focused on fleet fuel efficiency and alternative fuels rather than a reduction in passenger demand (International Air Transport Association 2021). Unfortunately, current technological improvements may only offer 1–2% improvements to fuel efficiency per year (Bows-Larkin 2015). Thus, Dolšak and Prakash (2022) argue in this journal that structural action is necessary to reduce aviation emissions, alongside individual lifestyle changes.

The European Union’s (EU) efforts towards climate action have broadly improved and expanded—with pauses—over time (Dupont and Torney 2021; Tobin and Schmidt 2021). Following the crisis-ridden decade of the 2010s, in which EU commitment to and capacity for climate action fluctuated (Bäckstrand and Elgström 2013; Burns et al. 2020; von Homeyer et al. 2021), the European Green Deal (EGD) firmly re-established the issue of climate change as a top priority for the EU. As part of the EGD, the EU has committed to becoming climate neutral by 2050, and seeks to secure emissions reductions across a range of economic sectors (European Commission 2019). For instance, the EGD calls for a 90% reduction (on 1990 levels) in transport emissions by 2050 (European Commission 2021). Achieving such a goal will be challenging, especially in the aviation sector: for example, in 2019, airline *Ryanair* was the only non-power plant within the top ten biggest emitters of the EU’s Emissions Trading System (ETS) (European Commission 2020). Yet, the political and economic turbulence caused by the COVID-19 pandemic led governments to protect the aviation sector and directly related jobs, with limited discussion of the need to curb aviation emissions (Russel 2020). Completed just as the pandemic was beginning, between late 2018 and mid-2020, Member States submitted National Energy and Climate Plans (NECPs). These NECPs are a key building block for achieving the goals of the EGD (European Commission 2019: 23). Thus, we ask: how frequently do EU Member States refer to aviation in their NECPs, and how can these references be understood thematically? This research question lays the groundwork for uncovering whether individual countries discuss national approaches to aviation and what are their policy preferences. The newly collected data may also become a useful starting point for further comparative studies in the future. By analysing how Member States engage with aviation in their NECPs, we create a ‘pre-pandemic baseline’ of countries’ stances towards this carbon-intensive sector. This baseline holds benefits for future research, as it enables analyses of on-going climate action strategies and the impact of the pandemic on this vital sector to be undertaken.

The remainder of the article is structured as follows. In the “Data and methods” section, we introduce our original NECP dataset and explain our two-stage summative content analysis approach, comprising first a content analysis, and then a thematic analysis. In the “Results” section, we present our findings for the two analytical stages, showing an increased quantity of references between the draft and final NECPs, but widely varying approaches to climate mitigation in the aviation sector between Member States. Finally, in the “Discussion and Outlook” section, we discuss our findings and propose that the NECPs provide an effective means of conducting cross-country comparison of sectoral climate mitigation planning by EU Member States.

## Data and methods

The NECPs are the most comprehensive climate-related documents to be created by EU Member States. The plans follow a pre-defined structure stipulated by the EU Energy Union Governance Regulation ((EU)2018/1999). As such, for example, the expansion of renewables is an obligation, while measures on lowering aviation emissions are not, and therefore are likely only to be included within more ambitious NECPs. Drafts of the NECPs were submitted to the Commission in late 2018, followed by finalised versions in late 2019 to mid-2020, following guidance from the Commission and lobbying from the public, media and NGOs. To date, the NECPs have received only limited academic attention, and the studies that exist have been based on single case studies, rather than comprehensive cross-country analysis. For example, Aboltins et al. (2020) analysed Latvia’s NECP, finding that in several sectors, more stringent measures would be needed. Meanwhile, Geissler et al. (2022) analysed the Austrian NECP regarding municipal spatial planning and investment in renewable energy sources. Thus, we see merit in conducting a comparative analysis of every NECP.

To answer our two-part research question, we analyse all draft and final NECPs using a two-stage ‘summative content analysis’ method (Hsieh and Shannon 2005). In stage 1, we count the keywords associated with aviation in each *draft* and *final* NECP. For the 26 NECPs available in English, we searched for 11 keywords.^1^ France’s draft NECP was available only in French, so we searched for 10 French-language keywords, which were then categorised with their English-language equivalents.^2^ We then averaged the totals in two ways: by the number of countries in each region to obtain a regional outlook; and by the number of references per 100 pages, to account for differences in the documents’ lengths. Finally, we compared states’ averages in two ways: by geographical region, as defined by EuroVoc^3^, to analyse regional patterns; and by date of EU accession, to explore whether the duration of EU membership influences policy development. While this stage of our analysis is effective for assessing and comparing the engagement of each Member State towards aviation in quantitative terms, it does not account for the nature of this engagement. Hence, we undertook a second, qualitative stage of analysis.

In stage 2, we inductively and iteratively developed *themes* and *sub-themes* for each sentence in the final NECPs that contained an aviation reference (*n* = 494).^4^ We analysed the final NECPs alone at this stage because these documents reflect each Member State’s finalised planning document after input from the European Commission. We coded each sentence and recoded previous documents whenever we identified a new theme. To ensure the reliability of this stage, each author coded a set of NECPs, and also reviewed a set that had been coded by another author, meaning that every reference to aviation was examined twice, and recoded if two coders agreed to do so. More detailed data on aviation references (stage 1) and themes/sub-themes is available in the Additional file 1.

## Results

### Stage 1: content analysis

In our content analysis of the *draft* NECPs, we found 238 aviation-related references, or 4.8 references per 100 pages (see Table 1). Austria was the only state that did not mention aviation, with few references from Romania (1), Finland (2) and Poland (2). France (33) and Spain (33) referenced aviation in their drafts the most frequently (see Additional file 1 for detailed data on aviation references by Member State and keyword). Regarding references per 100 pages, Luxembourg (12.3) and Spain (11.8) referenced aviation most frequently, followed closely by France and Sweden (11.4). Grouping the states geographically, the states in the Southern EuroVoc region referenced aviation the most on average per country, while Western region states mentioned aviation the most often per 100 pages (Table 1). On average, states that joined the EU before 2004 referenced aviation at nearly twice the rate as those that joined in 2004 or after.

**Table 1:** References to aviation in draft and final NECPs

	Average aviation references per country			Average aviation references per 100 pages		
**EuroVoc regions**	Draft	Final	*Change*	Draft	Final	*Change*
Central and Eastern (*n* = 8)	5.4	9.9	*83%*	2.6	3.3	*27%*
Northern (*n* = 6)	6.8	17.7	*160%*	5	8.8	*76%*
Southern (*n* = 6)	12.7	23.5	*85%*	5.9	7.6	*29%*
Western (*n* = 7)	11.1	32.9	*196%*	6.6	11.7	*77%*
**EU membership**	Draft	Final	*Change*	Draft	Final	*Change*
Before 2004 (*n* = 14)	11.6	27.2	*134%*	6.7	10	*49%*
2004 and after (*n* = 13)	5.8	13.5	*133%*	3	4.9	*63%*
**Overall (***n* = **27)**	8.8	20.6	*134%*	4.8	7.5	*56%*

The *final* NECPs showed a 134% increase in average aviation-related references *per country* compared to the drafts (see Additional file 1 for more details); there was almost no distinction between pre-2004 and 2004–onwards states in this regard. The combined length of the NECPs increased by 52% from 4930 to 7408 pages, accompanied by a 56% increase in references per 100 pages, suggesting increased engagement as states added pages to respond to feedback from the Commission. This increased engagement was not evenly distributed: the Central and Eastern states continued to make markedly fewer aviation references than the other three groups (see Table 1), while references in the pre-2004 membership group remained around twice as high as those of the 2004–onwards group. Belgium increased from 10 aviation references in its draft to 60 in its final NECP, representing the highest total and largest absolute increase, followed by France (44), Germany (42), Spain (42), and Sweden (39). The seven Western states increased their per-country references by 196%; more than double the percentage increase of Central and Southern states. Every state referenced aviation in their final NECPs, with Romania (2) including the fewest references overall, followed by Finland (3) and Portugal (3). On average, Sweden referenced aviation the most per 100 pages (19.6 times), while Romania was the lowest, at 0.9 references per 100 pages.

### Stage 2: thematic analysis

In stage 2 (see Table 2), we identified three main themes, which we divided into 17 sub-themes. The most common main theme was ‘contextual information’ (44% of total), comprising six sub-themes. Most commonly, ‘contextual information’ references described historical ‘energy demand/consumption’ in each state, for example the predicted trajectory of aviation’s growth, prior to the COVID-19 pandemic. As an example of such a reference, Bulgaria (2020:52)—a Central and Eastern Member State with lower GDP per capita that joined the EU in 2007—stated ‘[t]he overall increase in energy consumption in transport is attributable to the aviation segment, which is expected to grow by approximately 35% as compared to 2020’ by 2030. In contrast, for wealthier, Western European pre-2004 Member State Ireland, ‘[t]ransport energy demand (including aviation) is projected to fall year-on-year from 2021 to 2030 […] even though growth in transport activity demand is anticipated.’ Both quotations reflect anticipated growth in the transport sector but demonstrate the uneven trajectories of expected energy consumption levels between Western and CEE states. The second most common sub-theme within ‘contextual information’ described current or predicted levels of GHG emissions, and the third referred to figures that *excluded* aviation from the data. That is, 46 references to aviation within NECPs were explaining that aviation was excluded from the topic that was being described. For example, detailing a GHG emissions national transport target, Sweden (2020:16) specified ‘[a] special intermediate target for greenhouse gas emissions from national transport (except for national aviation, which belongs to the EU ETS) for 2030.’ The fourth sub-theme noted the prevalence of subsidies for fossil fuels, such as exemptions from excise duty for aviation fuels (Czechia, Denmark, Estonia, France, Italy, Latvia, Lithuania, Netherlands, Slovakia, Spain, and Sweden). The fifth contextual sub-theme simply described current and/or predicted passenger/freight figures, while the sixth sub-theme focused on interactions with renewable energy. Thus, the most common theme represented the description and/or forecasting of aviation activities, rather than pledges to reduce emissions.

**Table 2:** Aviation-related themes and sub-themes identified in final NECPs

Themes	Overall (*n* = 27)	Regions				EU membership	
		Central/Eastern (*n* = 8)	Northern (*n* = 6)	Southern (*n* = 6)	Western (*n* = 7)	Before 2004 (*n* = 14)	2004 and after (*n* = 13)
**Contextual information**	274	56	52	75	91	153	121
*Energy demand/consumption*	95	14	16	35	31	49	47
*Aviation greenhouse gas emissions*	73	17	18	12	26	37	36
*Exclusion of aviation-related emissions*	46	9	9	12	16	32	14
*Fossil fuel subsidies*	23	3	8	6	6	17	6
*Passenger/freight demand*	22	13	0	9	0	5	17
*Aviation/renewables interactions*	15	0	1	1	13	14	1
**Public policy**	213	23	39	43	108	169	44
*EU Emissions Trading System*	70	11	16	17	26	42	28
*Taxes*	37	0	8	2	27	37	0
*Airport-related mitigation*	33	3	3	15	12	27	6
*Other policy (net zero, emission plans, procurement)*	27	3	5	3	16	23	4
*Research and development*	22	3	6	4	9	19	3
*ICAO/CORSIA/carbon offsets*	20	2	0	2	16	18	2
*Reduce number of flights*	2	1	0	0	1	1	1
**Fuels/propulsion**	78	2	15	26	35	76	2
*Biofuels*	36	0	2	15	19	36	0
*Other fuels (hydrogen, synthetic, carbon free, other)*	17	0	4	5	8	17	0
*Energy/fuel efficiency*	15	2	3	4	6	13	2
*Electric/hybrid*	10	0	6	2	2	10	0
**Miscellaneous**	64	6	11	13	34	47	17

The second most common theme (34% of total) related to existing or planned public policies for reducing aviation emissions: Western states mentioned this theme the most frequently. The EU ETS was the most discussed public policy sub-theme, due to the fact that carbon dioxide emissions from flights within the EU have been included in that policy’s scope since 2012. References to the ETS were often descriptive and limited to discussing the inclusion of aviation emissions (e.g., Ireland 2020:95; Lithuania 2020:130). CEE states exhibited the fewest ETS sub-theme references to aviation. Aviation taxation was the second most frequent public policy sub-theme, but strikingly all references to taxation were made by states with pre-2004 EU membership. Spain’s (2020:276) and Italy’s (2020:156) NECPs explained how fossil fuels for aviation are usually not taxed. Austria’s (2020:111) NECP argued that taxing aviation tickets ‘can tempt passengers away from air travel’ and Belgium (2020:106) favoured an aviation tax reform ‘preferably harmonised at EU level […or] even at global level’ to finance the climate transition. Germany (2020:214) likewise supported a new tax regime, highlighting the benefits for other modes of transportation. Other sub-themes were more nationally focused, including mitigation-related actions at airports (e.g., Austria 2020:124), and plans for increased Research and Development (e.g., Sweden 2020:163). A smaller sub-theme comprised actions under the Carbon Offsetting and Reduction Scheme for International Aviation (CORSIA), a global market-based measure negotiated under the International Civil Aviation Organisation (ICAO). Under CORSIA, international airlines agreed to monitor emissions and offset 80% of those emitted above a 2020 baseline (European Commission 2021). Across all of the NECPs, there were only two references to reducing the number of flights, one each in the German and Croatian NECPs.

The third theme related to fuels and propulsion (12% of total). Such references argued that alternative fuels can help achieve decarbonisation in the aviation sector. For example, Austria (2020:67) stated that ‘the blending of alternative fuels should become mandatory in the aviation sector in the medium term’. Belgium (2020:205) also supported advanced biofuels and synthetic fuels to decrease aviation emissions. Sweden's (2020:117) and Portugal's (2020:45) NECPs described biofuels as a reliable bridging alternative in the transportation sector, decreasing petroleum reliance in the future. Spain’s (2020:94) NECP promoted the use of biofuels with specific consumption objectives as ‘the only way to reduce the use of fossil fuels over the coming years’. In sum, several Western and Southern states highlighted the potential benefits of alternative fuels, or showed support for increased research efforts, potentially reflecting the utility of the NECPs for agenda-setting, and also the reality that states would rather change fuels than reduce the total number of flights.

## Discussion and outlook

How frequently do EU Member States refer to aviation in their NECPs, and how can these references be understood thematically? By conducting a comprehensive analysis of engagement with aviation, several trends are apparent. Noteworthy is the 134% increase in average references to aviation per country in the final documents compared to the draft NECPs, suggesting the possible influence of the Commission’s feedback. Another key finding is the generally limited engagement with aviation from CEE states. Relatedly, states that joined the EU before 2004 referenced aviation twice as frequently as those that joined in 2004 or after, and had nearly twice as many references per 100 pages. References to aviation were split roughly evenly between contextual information and proposed solutions related to public policy and alternative fuels. The states focus much of their attention in the NECPs on market-based mechanisms, such as the EU ETS and taxes, as well as fuel substitution, amid marginal discussion of alternative options, such as reducing the number of flights, despite the importance of such changes (Dolšak and Prakash 2022).

Having established a baseline of NECP aviation engagement through this study, what may be the impact of the pandemic on the aviation sector and how could it inform policy making? Mobility restrictions and lockdowns during the pandemic may produce behavioural shifts, or simply lead to an urgent desire to travel overseas once again. Meanwhile, the rising cost of living in 2022 has not hastened a discussion about carbon taxes: only a few countries, notably Sweden, had already implemented such taxes for airplane tickets. While some NECPs mentioned future plans for tax measures, there are also indications that they will halt such efforts. For example, when the German government presented their climate package in late 2019, it stated that ‘[f]rom 2020, a higher flight tax will be introduced’ (Bundesregierung 2019). This tax was supposed to be implemented from April 2020 onwards, encouraging the use of trains over short domestic flights. However, such plans were put on hold during the pandemic, with Germany’s government bailing out Lufthansa without any binding agreements over climate protection (Russel 2020). Dupont and Torney (2021) found that the early recovery plans for COVID-19 showed accelerating efforts towards climate change and the implementation of the EGD. Indeed, regarding aviation, the French and Austrian governments’ bailouts of airline companies included conditionalities for some ‘greening’ measures. Yet, these conditionalities were isolated examples, with most governments supporting aviation without any additional climate requirements.

We propose several research avenues following our analysis of the aviation sector. First, taxation and changes to fuels have been previously identified as crucial to lowering aviation emissions (Larsson et al. 2019). Regarding the ‘taxation’ sub-theme and the ‘fuels’ theme, we find that Western EU states discussed new steps the most, followed by Northern states for taxation and Southern states for biofuels. CEE states did not mention taxation and only twice referenced fuels in any way. Since the EU ETS has clear guidelines towards the treatment of aviation, why was there such a breadth in approaches between the NECPs? We suggest some early hypotheses for future research. One explanation for the lack of a uniform approach may be that countries with lower income per capita, for example, CEE states, actively seek to increase trade and tourism, while their higher-income Western counterparts, by tentatively proposing some steps beyond what is required under the EU ETS, may aim to increase and foster research and business opportunities that stem from alternative fuels. Another explanation may be that the borderless nature of aviation and the fact that the Paris Agreement excluded aviation emissions have ensured that national governments feel less pressure to act in this sector. Future studies could also focus on the airline industry, their research on and stake in alternative fuels, and whether they were involved in the NECP process. Such research could give insights into whether they have significant agenda-setting power in national-level climate-related planning.

Concluding, we argue that the NECPs provide an effective means of obtaining a broad overview of states’ positions on sectoral climate issues, which can then be used as a starting point for more fine-grained analysis. Our analysis of the NECPs shows that prior to the turbulence of the COVID-19 pandemic, there was a baseline of relatively low attention to aviation, particularly within newer Member States. Thus far, government responses to the pandemic suggest that states are not doing enough to ensure that the aviation sector is on track to contribute to the EU’s transformation into a climate neutral continent.

## Supplementary Information

**Additional file 1.** Aviation NECP thematic analysis.

## Data Availability

Made available and will be stored in a data repository.

## Footnotes

1 “aeronautic*”, “aeroplane*”, “aerospace”, “aircraft*”, “airplane*”, “airport*”, “aviation”, "air transport", "air travel", “flight*” and “flying”. We excluded non-aviation-related references to flight.

2 aéronautique, aéroplane, aérospatiale, aéronef, avion, aéroport, aviation, vol, transport aérien, aérien.

3 There are four major physical regions defined by the EU: Northern Europe, Western Europe, Central and Eastern Europe, and Southern Europe, with each subregion comprising several countries.

4 Approximately 5% of coded sentences (*n* = 27) contained multiple aviation references.
